# Recommendations for the formatting of Variant Call Format (VCF) files to make plant genotyping data FAIR

**DOI:** 10.12688/f1000research.109080.2

**Published:** 2022-05-19

**Authors:** Sebastian Beier, Anne Fiebig, Cyril Pommier, Isuru Liyanage, Matthias Lange, Paul J. Kersey, Stephan Weise, Richard Finkers, Baron Koylass, Timothee Cezard, Mélanie Courtot, Bruno Contreras-Moreira, Guy Naamati, Sarah Dyer, Uwe Scholz

**Affiliations:** 1 Breeding Research, Leibniz Institute of Plant Genetics and Crop Plant Research (IPK) Gatersleben, Seeland, 06466, Germany; 2 Institute of Bio- and Geosciences, Bioinformatics (IBG-4), Forschungszentrum Jülich GmbH, Jülich, 52425, Germany; 3 BioinfOmics, Plant bioinformatics facility, Université Paris-Saclay, INRAE, Versailles, France; 4 European Molecular Biology Laboratory, European Bioinformatics Institute, Hinxton, UK; 5 Royal Botanic Gardens, Kew, Richmond, UK; 6 Plant Breeding, Wageningen University & Research, Wageningen, The Netherlands; 7 Gennovation B.V., Wageningen, The Netherlands; 8 Ontario Institute for Cancer Research, Toronto, Canada; 9 Laboratorio de Biología Computacional y Estructural, Estación Experimental Aula Dei-CSIC, Zaragoza, 50059, Spain

**Keywords:** FAIR, plant, genotyping, snp, vcf, data management, phenotyping, ELIXIR

## Abstract

In this opinion article, we discuss the formatting of files from (plant) genotyping studies, in particular the formatting of metadata in Variant Call Format (VCF) files. The flexibility of the VCF format specification facilitates its use as a generic interchange format across domains but can lead to inconsistency between files in the presentation of metadata. To enable fully autonomous machine actionable data flow, generic elements need to be further specified.

We strongly support the merits of the FAIR principles and see the need to facilitate them also through technical implementation specifications. They form a basis for the proposed VCF extensions here. We have learned from the existing application of VCF that the definition of relevant metadata using controlled standards, vocabulary and the consistent use of cross-references via resolvable identifiers (machine-readable) are particularly necessary and propose their encoding.

VCF is an established standard for the exchange and publication of genotyping data. Other data formats are also used to capture variant data (for example, the HapMap and the gVCF formats), but none currently have the reach of VCF. For the sake of simplicity, we will only discuss VCF and our recommendations for its use, but these recommendations could also be applied to gVCF. However, the part of the VCF standard relating to metadata (as opposed to the actual variant calls) defines a syntactic format but no vocabulary, unique identifier or recommended content. In practice, often only sparse descriptive metadata is included. When descriptive metadata is provided, proprietary metadata fields are frequently added that have not been agreed upon within the community which may limit long-term and comprehensive interoperability. To address this, we propose recommendations for supplying and encoding metadata, focusing on use cases from plant sciences. We expect there to be overlap, but also divergence, with the needs of other domains.

## Introduction

As of today, there are several public repositories for genetic and genomic variation data. However, most of these repositories are exclusive to humans and do not include other organisms ( Cezard *et al.*, 2021), such as dbSNP ( Sherry *et al*., 2001), dbGaP ( Mailman *et al*., 2007) and dbVar ( Lappalainen *et al*., 2013). There are two main resources for non-human variation data: The European Variation Archive (EVA) ( Cezard *et al*., 2021), hosted by EMBL-EBI, and the Genome Variation Map (GVM) ( Song *et al*., 2018), hosted by CNCB-NGDC. Submitting datasets to these two repositories works very similarly, but we will focus on the submission of genotyping datasets to EVA. Data and metadata are submitted to a File Transfer Protocol (FTP) file server and, after a quality check, are added to the database and displayed on their respective websites or kept hidden until a user-specified release date. Data are only checked for a few critical points: first, the VCF file must comply with the Variant Call Format (VCF) ( Danecek *et al*., 2011) specifications, second, the genome assembly used as reference must be registered with one of the databases of the International Nucleotide Sequence Database Collaboration (INSDC) ( Cochrane *et al*., 2011), i.e., GenBank ( Benson *et al*., 2013), the European Nucleotide Archive (ENA) ( Leinonen *et al*., 2011) or the DNA Data Bank of Japan (DDBJ) ( Mashima *et al*., 2017), respectively, and an accession number is available, and third, the VCF file must contain either allele frequencies and/or genotype information.

When a data submission is made to the EVA, samples are automatically registered in the associated BioSamples database ( Courtot *et al*., 2022), unless this has been explicitly done previously by the data submitter. Such automatically created samples possess only the minimum necessary attributes (name, domain, release date) and no other descriptive metadata. If pre-registering samples in BioSamples, metadata can be specified as key-value pairs. For some specific use cases, there are already predefined checklists that list which metadata should be supplied on sample registration, against which the metadata can be validated. Additional information, which is not yet available in a defined attribute, can also be submitted under a free text key. We recommend the manual registration of samples at BioSamples as this gives the greatest flexibility when editing and adding information.

The description of these samples must take into account plant specificities to fully enable findability and interoperability with reference databases such as EURISCO ( Weise *et al.*, 2017) or datasets like phenotyping experiments to allow for complete reusability. The biggest challenge is probably the accurate identification of biological material, including varieties, lines, RILs, and crosses. This has been standardised by the MCPD ( Alercia *et al.*, 2015) and MIAPPE ( Papoutsoglou *et al.*, 2020) data standards, which introduce an international ID mechanism based on DOI and handling of genealogy and pedigree. Indeed, experience has shown that the mere indication of a cultivar name is not sufficient and introduces a lot of ambiguities regarding the material that has actually been genotyped.

One hurdle in enforcing a uniform format specification for variation data in plant science is the fact that there are some databases that offer their own plant-specific variation data. These databases often belong to either the project-specific or the aggregation database classes (which are often organism-specific). The former do not change after the project lifetime, while the latter summarise the results of several studies in a uniform way. Probably the best known project-specific database is the GMI-MPI of the 1001 Genomes Consortium ( Alonso-Blanco *et al.*, 2016). An example for an aggregation database would be https://jbrowse.arabidopsis.org where a large number of *Arabidopsis Thaliana* VCF records from different studies is stored. In general, it should be possible to adapt metadata to new guidelines for project-specific databases, whereas it would be much more difficult to obtain this information for aggregation databases.

Another useful resource for the analysis of plant variation data is Ensembl Plants ( Howe *et al*., 2020). This database, also hosted by EMBL-EBI, is a platform for displaying and visualising plant genomes. If the reference genome assembly associated with the data submitted to EVA is supported in Ensembl, then it is possible to display genetic variants in their genomic context in the Ensembl browser, each linked to its sample. Data submitters should contact Ensembl helpdesk to request it. VCFs in EVA should be available as browsable files, as seen for example in soybean.

## Lessons learned from studies on plant phenotyping and its application to metadata information in genotyping

The standardisation of plant variation data is still in its infancy. Therefore, it is beneficial to look to other data types for guidance and improvement. One particular data type where a lot of standardisation work has been done in recent years is plant phenotyping. Plant phenotyping has developed rapidly with the introduction of high-throughput technologies such as fully automated greenhouses, full-time sensor recording and aerial observation drones. The need to record data points and the method of observation has led the community to implement a standard for describing such experiments: MIAPPE ( Papoutsoglou *et al*., 2020) (Minimal Information About a Plant Phenotyping Experiment). Since its introduction in 2014, the standard has been extended to describe sample material (including the anatomical part sampled) through the use of specialised ontologies. MIAPPE-compliant data can be represented in the Investigation-Study-Assay (ISA) framework for structured data representation ( Rocca-Serra *et al*., 2010) and exposed programmatically via the Breeding API (BrAPI) ( Selby *et al*., 2019). The format is maintained and regularly updated by an active community. Fully MIAPPE-compliant data is rich in metadata that describes and identifies in detail both the sample material and the experiment performed. One aim is to allow machine access to the data via application programming interfaces (APIs). Therefore, the use of controlled vocabularies is encouraged by supporting different ontologies, with AgroPortal ( Jonquet *et al*., 2018) serving as a reference repository.

In contrast, genotyping data is often published and shared without sufficient metadata to ensure interoperability and reuse, as seen with other data formats ( Bernstein *et al*., 2017). Current automated tools do not fill in the metadata fields very well, leaving the user to take care of it. Some information that should be recorded cannot be easily retrieved from the analysis results, such as the identification of biological material studied, or the reference genome assembly and version used. Depending on who is handling the data and what skills are associated with the role, the difficulty of providing well-formatted metadata will vary. Bioinformaticians who have directly performed the genotyping analyses and thus the creation of the VCF files will consider it a comparatively simple task to enter metadata directly into the file. Similarly, a data steward who may not have previously been directly familiar with the data but with the structure itself should have no problems. Gathering experimental data from conversations with wet lab colleagues or in a laboratory information management system (LIMS) search will be the more laborious activity for individuals in either role. However, experimentalists who have little or no experience with the required metadata formats are most likely to be overwhelmed without a simple GUI or input template. Principal investigators who want to submit the data at the end of an experiment may have similar difficulties. From these observations, there is an urgent need for supporting tools or APIs for the structural and content validation of VCFs. We recommend performing both metadata and data validation. For the validation of VCF files, we recommend EBI’s VCF validator.

## Data and metadata formatting

**Figure 1.**
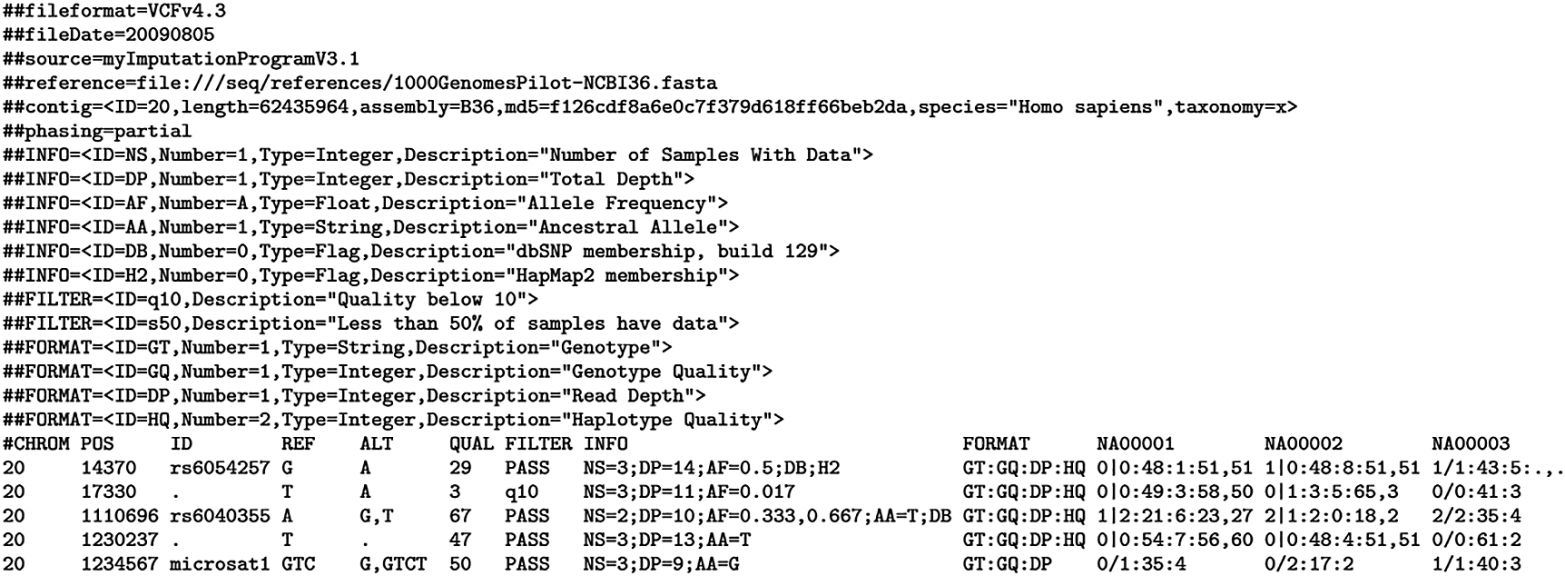
Example Variant Call Format (VCF) file structure, including meta-information lines and data lines (from https://samtools.github.io/hts-specs/VCFv4.3.pdf).

The *de facto* standard data format for genotyping studies is the Variant Call Format (VCF). The following statements are based on the current version 4.3. A VCF file comprises a single text file that consists of three parts: (i) one or more meta-information lines, initiating with a ## describing the settings, samples and general experimental design of the genotyping study. File meta-information is included after the ## string and must be key=value pairs. There are currently no guidelines on how these are used or what they may contain (ii) a header line initiating with a single #, and (iii) one or more data lines, each recording the genotype calls at each varying position in the reference genome assembly for a single sample. Both the header and data lines use tab stops to delineate separate fields. Meta-information lines are considered optional; however, they need to be well-formed if present. This means that all structured lines that have their value enclosed within “<>” require an ID which must be unique within their type ( Figure 1).

A critical aspect of VCF specifications is that sample naming within the VCF file does not follow any standard specifications, i.e. users can name their samples without reference to any real biological material. Even worse, phenotyping and genotyping data from the same experimental setup often use different sample identifiers even when the same biological material has been used, which makes it difficult to reconstruct later which datasets were derived from a common sample. To be able to represent such relationships, descriptive metadata is required that relates these different sample identifiers to each other.

In response to the points discussed previously, we propose a minimal list of metadata fields, recommend an identifier schema and guidelines for vocabulary and data format within a VCF file. Our suggestions are divided into recommended and optional changes. Although, we are primarily addressing data submissions to the EMBL-EBI repositories BioSamples and EVA (and implicitly ENA through the submission of sequence information), subsequent formatting guidelines should be applied regardless of the specific deposition repository and should also be considered when designing databases and APIs.

In our view, these additional fields should be required for a valid VCF:

One meta-information line, ##fileformat, is obligatory in VCF. We also recommend using the additional lines ##filedate, ##bioinformatics_source, ##reference_ac, ##reference_url, ##contig and ##SAMPLE. To ensure permanent unique and stable IDs for samples and genotypes, we recommend the registration of used genotypes and samples in the BioSamples database. This enables the publishing of biological material used in variation studies, and we explicitly recommend the use of long-term stable BioSamples identifiers as primary IDs for material description in VCF files ( Table 1).

**Table 1.** Summary of recommendations for metadata formatting.

Metadata field	Definition	Format	Example	Cardinality
##fileDate	Creation date of the VCF file	Date (ISO 8601, YYYYMMDD)	##fileDate=20120921	1
##bioinformatics_source	Chains of bioinformatics tools for creating the VCF file	URL, DOI	##bioinformatics_source=“ doi.org/10.1038/s41588-018-0266-x”	1
##reference_ac	Accession number of reference genome assembly used in the VCF file	/[(GCA/GCF)_(d){9}\.(0-9)*]/	##reference_ac=GCA_902498975.1	1
##reference_url	URL of the reference genome assembly used in the VCF file	URL, DOI	##reference_url=“ ftp.ncbi.nlm.nih.gov/genomes/all/GCA/902/498/975/GCA_902498975.1_Morex_v2.0/GCA_902498975.1_Morex_v2.0_genomic.fna.gz”	1
##contig	Metadata about a single sequence in the reference genome assembly	Composite (see below)	##contig=<ID=chr1H,length=522466905,assembly=GCA_902498975.1,md5=8d21a35cc68340ecf40e2a8dec9428fa,species=NCBITaxon:4513>	1:N
	The primary identifier of the sequence	String	ID=chr1H	1
	The length in base pairs (bp) of the sequence	Integer	length=522466905	1
	The assembly accession number this sequence belongs to	/[(GCA/GCF)_(d){9}\.(0-9)*]/	assembly=GCA_902498975.1	1
	The md5 checksum of the sequence	MD5	md5=8d21a35cc68340ecf40e2a8dec9428fa	1
	The species of the sequence (NCBI Taxon ID)	/[(NCBITaxon):(\d+)]/	species=NCBITaxon:4513	1
##SAMPLE	Metadata about a single sample genotype that is part of the genotyping experiment in the VCF file	Composite (see below)	##SAMPLE=<ID=SAMEA104646767,DOI=“ doi.org/10.25642/IPK/GBIS/7811152”>	1:N
	The primary identifier (BioSamples Database identifier) of the genotyping sample	/[(SAM)(E|N|D)(A|G)(\d+)]/	ID=SAMEA104646767	1
	The DOI of the genotyping sample (if available)	URL, DOI	DOI=“ doi.org/10.25642/IPK/GBIS/7811152”	0-1
	The external identifiers under which this genotyping sample is registered in other databases (either ‘FAO-WIEWS_instcode:genus:accession_number’ or ‘DNS:database_identifier:identifier_scheme:identifier’)	See Definition	ext_ID=“DEU146:Hordeum:HOR 1361 BRG” or ext_ID=“ipk-gatersleben.de:GBIS:akzessionId:7811152”	0:N

### File date field format

The creation date of the VCF should be specified in the metadata via the field ##fileDate, the notation corresponds to ISO 8601 ( Kuhn, 1995) (in the basic form without separator: YYYYMMDD).

##fileDate=date

Example:

Description of a VCF file that was created on September 21st in 2012.

##fileDate=20120921


### Bioinformatics source field format

The analytic approach (usually consisting of chains of bioinformatics tools) for creating the VCF file is specified in the ##bioinformatics_source field. Such approaches often involve several steps, like read mapping, variant calling and imputation, each carried out using a different program. Every component of this process should be clearly described, including all the parameter values.

##bioinformatics_source=url

This is ideally specified as the DOI of a publication, or more generally as URL/URI (like a public repository for the scripts and parameters used).

Examples:

1) Description of a GBS experiment in barley and subsequent read alignment and variant calling using a bioinformatics analysis pipeline consisting of cutadapt, BWA-MEM, SAMtools, NovoSort, Picard, BCFtools and seqArray.


##bioinformatics_source="doi.org/10.1038/s41588-018-0266-x"

2) Modified version of Tassel4 (v.4.3.7) for running the Tassel-GBS pipeline modified for polyploid species with high read depths used in ( Pereira *et al*., 2018).


##bioinformatics_source="github.com/gramarga/tassel4-poly"


### Reference_ac field format

This field contains the accession number (including the version) of the reference sequence on which the variation data of the present VCF is based.

##reference_ac=assembly_accession

The NCBI page on the Genome Assembly Model states ( NCBI, 2002): “The assembly accession starts with a three letter prefix, GCA for GenBank assemblies […]. This is followed by an underscore and 9 digits. A version is then added to the accession. For example, the assembly accession for the GenBank version of the public human reference assembly (GRCh38.p11) is GCA_000001405.26”. Note these accessions are shared by all INSDC archives.

Example:

Reference genome assembly for barley ( *Hordeum vulgare*) cultivar Morex version 2.



##reference_ac=GCA_902498975.1


### Reference_url field format

While the ##reference_ac field contains the accession number of the reference genome assembly, the ##reference_url field contains a URL (or URI/DOI) for downloading of this reference genome assembly, preferably from one INSDC archive.

##reference_url=url

The reference genome assembly should be in FASTA format; the user is free to provide a packed or unpacked publicly available version of the genome assembly.

Example:

Reference genome assembly for barley ( *Hordeum vulgare*) cultivar Morex version 2 download link on NCBI FTP.



##reference_url=“ftp.ncbi.nlm.nih.gov/genomes/all/GCA/902/498/975/GCA_902498975.1_Morex_v2.0/GCA_902498975.1_Morex_v2.0_genomic.fna.gz”


### Contig field format

The individual sequence(s) of the reference genome assembly are described in more detail in the #contig field(s).

##contig=<ID=ctg1, length=sequence_length, assembly=gca_accession, md5=md5_hash, species=NCBI Taxon ID>

Each contig entry contains at least the attribute ID, and typically also include length, assembly, md5 and species. The ID is the identifier of the sequence contig used in the reference genome assembly. Length contains the base pair length of the sequence contig in the reference genome assembly. The assembly is the accession number of the reference genome. If the md5 parameter is given, please note that the individual sequence contigs MD5 checksum is expected, not the MD5 sum of the complete reference genome assembly. The species is the taxonomic name of the species of the reference genome assembly.

Examples:

1) Chromosome 1H of barley ( *Hordeum vulgare*) cultivar Morex version 2.


##contig=<ID=chr1H,length=522466905,assembly=GCA_902498975.1,md5=8d21a35cc68340ecf40e2a8dec9428fa,species=NCBITaxon:4513>

2) Chromosome 1 of maize ( *Zea mays*) cultivar B73 version 3.


##contig=<ID=GK000031.3,length=301433382,assembly=GCA_000005005.5,md5=74dfe85ad898416814fa98e8d7048f76,species=NCBITaxon:4577>


### Sample field format

The ##SAMPLE fields describe the material whose variants are given in the genotype call columns in greater detail and can be extended using the specifications of the VCF format.

##SAMPLE=<ID=BioSample_accession, DOI=doi, ext_ID=registry:identifier>

Genotyped samples are indicated in the VCF by the BioSample accession, which is formed as follows (based on information from the BioSamples documentation): “BioSample accessions always begin with SAM. The next letter is either E or N or D depending if the sample information was originally submitted to EMBL-EBI or NCBI or DDBJ, respectively. After that, there may be an A or a G to denote an Assay sample or a Group of samples. Finally, there is a numeric component that may or may not be zero-padded.” Additional information (like complete Multi-Crop Passport Descriptor ( Alercia *et al*., 2015) records) on the sample material is provided under the DOI ( Alercia *et al*., 2018). If there are additional IDs like project or database IDs, they can be provided alongside the DOI as “ext_ID”. They are strongly recommended if no DOI is available. If the material is held by a FAO-WIEWS recognised institution, the external ID consists of the FAO-WIEWS instcode, the genus and the accession number (see example 2). If the database is not registered with FAO-WIEWS, the DNS of the holding institution or laboratory, the database identifier, the identifier scheme and the identifier value should be provided (see example 3). For multiple external IDs the field should be used multiple times (delimited by commas). By default, the registry in the “ext_ID” field should follow the specification in Identifier.org according to MIRIAM ( Juty *et al.*, 2012).

Examples (Please note that all examples here represent the same genotype. To avoid misunderstandings, if available, the preferred method of describing the data is by DOI.):

1) One genotype from the barley ( *Hordeum vulgare*) GBS experiment with a DOI registered.


##SAMPLE=<ID=SAMEA104646767,DOI="doi.org/10.25642/IPK/GBIS/7811152">

2) One genotype from the barley ( *Hordeum vulgare*) GBS experiment with the FAO-WIEWS code available but no DOI.


##SAMPLE=<ID=SAMEA104646767,ext_ID="DEU146:Hordeum:HOR 1361 BRG">

3) One genotype from the barley ( *Hordeum vulgare*) GBS experiment with no DOI and no FAO-WIEWS code available.


##SAMPLE=<ID=SAMEA104646767,ext_ID="ipk-gatersleben.de:GBIS:akzessionId:7811152">


### Recommendations for data fields

In order to allow the highest degree of interoperability, we suggest using BioSamples IDs as the column headers for each sample. In the header line, they should be provided after the 9 mandatory column headings (#CHROM, POS, ID, REF, ALT, QUAL, FILTER, INFO, FORMAT).

In addition, ensure that the genomic positions in the data lines (consisting of the #CHROM and POS tuple) use the same nomenclature as in the reference genome assembly FASTA file and that the positions of the variations are within the start and end positions of the respective chromosome or contig. Watch out for programmes that change these values automatically (especially during imputation).

### Additional meta-information fields

On top of the preceding recommendations to improve findability, interoperability and reusability, we encourage everyone to describe their data in as much detail as possible in the metainformation lines. Before introducing new fields, please check the official format specifications (in VCFv4.3 this would be under 1.4 Meta-information lines) to avoid redundancy and possible incompatibilities.

## Conclusion

With the data and metadata recommendations for VCF files presented here, we hope to make a contribution to linking genotypic and other data for plants (e.g. phenotypic, transcriptomic, metabolomic data sets that can be linked through precise sample identifiers provided by BioSamples). In our view, the minimum to achieve this is to have traceable material and sample management. Analytical results should be linked out to the respective sample(s) and defined in the context of the study being reported by using the persistent BioSamples identifiers throughout all analytical results. One way to ensure this is to generate long-term stable identifiers at an early stage, ideally when the sample is taken, and to document all work steps accurately. Reproducibility is also an important aspect, which has recently been criticised more frequently in various studies ( Baker, 2016; Miyakawa, 2020). Technologies such as containers or the provision of the entire data set and the analytical computing pipeline in a cloud environment could be a further step towards overcoming such problems ( Grüning *et al*., 2018).

The BioSamples database at EMBL-EBI stores samples metadata and allows their pre-registration; it provides unique, stable identifiers for each sample. BioSamples connects to other archives, enabling consistent tracking through time and assays of the samples and derived data. It supports validation of plant metadata according to the MIAPPE standard, ensuring data FAIRness ( Wilkinson *et al*., 2016) at submission time as well as keeping metadata on hold pending publication of results. It is recognised by ELIXIR ( Harrow *et al.*, 2021) as a recommended Deposition Database for Biomolecular Data. This ensures that comprehensive, validated metadata can be captured at all stages of sample and data generation and that relationships between samples and derived data can be tracked across molecular archives.

There are several ways in which the recommendations published here can find acceptance in the larger plant science community, each with its advantages and disadvantages ( Sielemann *et al.*, 2020). By working with BioSamples, Ensembl plants and EVA, we are introducing these ideas to the plant science community at a key point during the data submission process. This will not change how smaller project or organism-based databases or variation data providers will operate, but hopefully can stimulate discussions about a higher level of FAIR for variation data.

One approach to enforcing these recommendations on a larger scale would be to contact the major publishers with a critical mass of supporters and ask them to consider these recommendations as a prerequisite for submitting new manuscripts. In addition, one could also approach various communities of plant scientists who would commit to following these recommendations without further outside influence, similar to the adaptation of the FAIR principles. Both options are very time-consuming and labour-intensive, although the second option has tended to prevail in the past. Especially with regard to the reusability of the data and the possibilities to combine it with new questions, it offers the greatest incentive to be positive about such a broad introduction.

**Figure 2.**
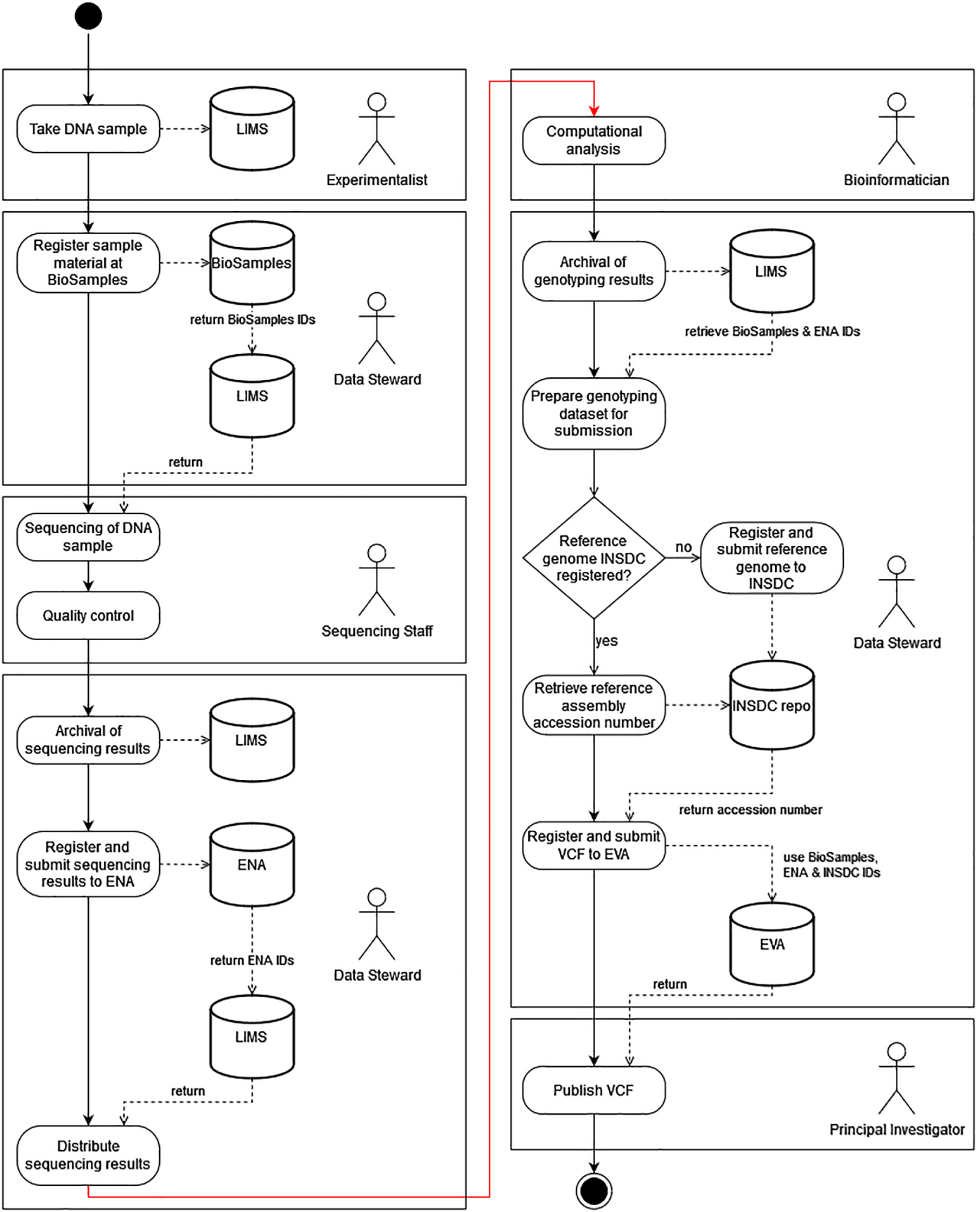
The recommended workflow for the submission of genotypic data to public EMBL-EBI databases. DNA samples are collected by an Experimentalist and their metadata are stored in a Laboratory Information Management System (LIMS). The Data Steward then registers these samples with BioSamples and in return receives unique BioSamples IDs back, which the Data Steward adds to the created samples in the LIMS. The sequencing and quality control of these samples is then carried out by the Sequencing Staff and the primary sequence data is fed into the LIMS and linked to the sample data by the Data Steward. The sequencing results are then registered and submitted to the European Nucleotide Archive (ENA) using the BioSamples IDs to link the initially submitted samples to the generated sequencing reads. The study identifiers (ENA IDs) are assigned by ENA and added to the samples by the Data Steward in LIMS. The Bioinformatician then analyses the data and produces the genotyping results. Afterwards, the Data Steward prepares these data for transmission by linking them to the already created sample data from the LIMS and extracting the required metadata and adding it to the header of the Variant Call Format (VCF) file. If the reference genome used for genotyping is not yet available in public repositories, it will now be transferred by the Data Steward to one of the International Nucleotide Sequence Database Collaboration (INSDC) databases. Otherwise, the metadata-enriched VCF file can be registered and submitted to the European Variation Archive (EVA). The identifiers assigned by EVA are then transmitted back and the Principal Investigator can approve the publication of the data.

The responsibilities of the people involved may vary from research institution to research institution, but the general tasks for the generation of plant genotyping data and the subsequent publication of these data follow a common pattern. To highlight how the complete data management of a genotyping project could be structured, we have designed an exemplary Unified Modeling Language (UML) diagram ( Figure 2) as a best practice proposal. We assume that the research institution has a LIMS and that sample collection, sample preparation, sequencing and all bioinformatic analyses are carried out in house. Even if one or more of these activities are outsourced, most data management activities (indicated in the figure by the actor “Data Steward”) and thus also the primary communication with public repositories remain the scientific responsibility of the research institution ( Mayer *et al.*, 2021). It is relatively obvious that the timing of interaction with public repositories varies greatly depending on the purpose (registration of datasets, retrieval of identifiers, or updating of datasets) and is recommended to occur at the earliest possible date in order to use the persistent identifiers of the datasets in the further course of the analyses and thus avoid errors due to the use of short-lived internal identifiers.

This approach to data management facilitates the submission of data for publication or at the end of the research project. Here, the situation often arises that the data steward, under time pressure, fails to submit the necessary (meta-)information to the public repositories. The submitted dataset therefore only consists of very generic and not meaningful metadata ( Toczydlowski *et al*., 2021). Such behaviour is the lesser evil compared to not publishing the dataset but can hinder its interoperability and reusability. During the peer review process, large and complex datasets often cannot be checked in depth by the reviewers. A wider use of automatic validations or checklists (such as those supported by BioSamples) that the metadata adhere to would enable reviewers and users to identify well-annotated datasets suitable for re-use.

The FAIRness of datasets improves when the metadata fields defined here are collected and submitted. Indeed, findability is increased by the recommended persistent identifiers, which allow search engines to use this information to find linked datasets. For example, plant material and sample identifications, as recommended here, are used as germplasm filters in the FAIDARE search portal, allowing discovery of genotyping and phenotyping data containing the same plant material. The accessibility of datasets remains unchanged, but interoperability is significantly improved. Indeed, the improved identification of common IDs for plant material through the use of the BioSamples infrastructure makes it possible to link and integrate distributed and heterogeneous datasets. Thanks to this facilitated interoperability, reusability through new analyses is also improved. For the stepwise FAIRification of a plant variant dataset, a recipe has been provided in the FAIR Cookbook that implements the recommendations presented here ( https://w3id.org/faircookbook/FCB061). Adoption of these guidelines and best practices will help make plant genotyping data FAIR and provide new opportunities to advance our understanding of relationships between genotypic and phenotypic data.

## Data availability

An example VCF conforming to the metadata recommendations presented here and comprising a barley genotyping experiment with 22626 accessions has been deposited at EVA under PRJEB51851 and is accessible via the study browser: https://www.ebi.ac.uk/eva/?eva-study=PRJEB51851.

See the FAIR Cookbook under the recipe https://w3id.org/faircookbook/FCB061 for step-by-step instructions on how to submit data according to the recommendations in this manuscript.
