# Supplementary material for: Recommendations for the formatting of Variant Call Format (VCF) files to make plant genotyping data FAIR
Source: F1000Res. 2022 May 19;11:ELIXIR-231. Originally published 2022 Feb 24. [Version 2] doi: 10.12688/f1000research.109080.2 (PMC9218589; doi:10.12688/f1000research.109080.2)
Supplement: Peer review history

# Peer review: Reviewer response for version 2

**DOI:** 10.5256/f1000research.134164.r138364

**Published:** 2022-06-22

**Authors:** Boas Pucker, Alenka Hafner

**Affiliations:** 1 Institute of Plant Biology & BRICS, TU Braunschweig, Braunschweig, Germany; 2 Intercollege Graduate Degree Program in Plant Biology, Penn State University, University Park, PA, USA

The authors addressed all our comments and the revisions are appropriate.

Is the topic of the opinion article discussed accurately in the context of the current literature?

Yes

Are arguments sufficiently supported by evidence from the published literature?

Yes

Are all factual statements correct and adequately supported by citations?

Yes

Are the conclusions drawn balanced and justified on the basis of the presented arguments?

Yes

Reviewer Expertise:

Plant genomics, plant specialized metabolism

We confirm that we have read this submission and believe that we have an appropriate level of expertise to confirm that it is of an acceptable scientific standard.

# Peer review: Reviewer response for version 2

**DOI:** 10.5256/f1000research.134164.r138365

**Published:** 2022-06-06

**Authors:** Micha M. Bayer

**Affiliations:** 1 Information & Computational Sciences (ICS) group, James Hutton Institute, Dundee, UK

I am happy with both the responses to my comments and the changes that have been made to the manuscript and will recommend final acceptance of the paper. I'd also like to thank the authors for this very useful contribution to the plant bioinformatics community.

Is the topic of the opinion article discussed accurately in the context of the current literature?

Yes

Are arguments sufficiently supported by evidence from the published literature?

Yes

Are all factual statements correct and adequately supported by citations?

Yes

Are the conclusions drawn balanced and justified on the basis of the presented arguments?

Yes

Reviewer Expertise:

Crop plant bioinformatics, variomics, genomics, transcriptomics

I confirm that I have read this submission and believe that I have an appropriate level of expertise to confirm that it is of an acceptable scientific standard.

# Peer review: Reviewer response for version 1

**DOI:** 10.5256/f1000research.120539.r128674

**Published:** 2022-04-19

**Authors:** Micha M. Bayer

**Affiliations:** 1 Information & Computational Sciences (ICS) group, James Hutton Institute, Dundee, UK

In this opinion article, Beier and co-authors review the current procedures for submitting genotypic variation data to public archives and make a number of recommendations to improve the standardisation of submitted data in order to advance the FAIR principles in this area. They show with specific examples how metadata in VCF files could be improved and suggest additional fields that should be included in VCF-based data submissions.

 This is a very useful paper that should help stimulate further discussion and progress in this area. The presentation is systematic, clear, and thorough. I have a number of suggestions for improvement that should be implemented before indexing, but otherwise, I am very happy to recommend indexing. This will be a very useful contribution to the community.

- What are the specific differences between plants and other organisms in this context? What requirements does plant data have that other data doesn’t? It would be good to expand on this a little.
- Should a standard such as MIAPPE be established for genotyping experiments/data? This would be a good place to discuss this and perhaps start describing what it could look like.
- The age of pan-genomes is now firmly upon us, and it might be a good idea to add some thoughts on how graph-based genotyping might affect any of the suggestions proposed here - for example, graph-based reference genomes.
- “ *Bioinformaticians who have directly performed the genotyping analyses and thus the creation of the VCF files will consider it a comparatively simple task to enter metadata directly into the file*.” It would be helpful to specify what tools can be used (and how) to add metadata to VCF headers and how this can be done with minimal risk of getting things wrong (e.g. mislabelling samples). Is manual editing of a header and replacing it with bcftools the best we have, or are there tools out there that can achieve this in a more foolproof/refined fashion?
- It would be beneficial to mention the ##META header lines for defining phenotype metadata that were introduced in the VCF v4.3 revision and to provide some examples of how they could be used. How do they fit into the existing framework of data in EVA and ENSEMBL?
- P.7, section “Sample field format”: Please include some detail on what the “registry” entails – is there a standardised way of creating/naming/referring to registries?
- P.9, bottom: Please provide a reference and/or URL for ELIXIR in the text here.
- Figure 2 is an idealised scenario that assumes an institution has both a LIMS and a data steward. Neither is a given – this very much depends on the organisational structure and the levels of funding available to an institution. It would be helpful to have – in addition – an alternative workflow that is more realistic/flexible and describes what can be done when these resources aren’t available.
- Figure 2: Replace pronouns like “he” with gender-neutral equivalents like “they” or use the passive voice.
- Figure 2: The header says “ *…submission of genotypic data to public databases*” but then the legend only mentions the EVA specifically.

Is the topic of the opinion article discussed accurately in the context of the current literature?

Yes

Are arguments sufficiently supported by evidence from the published literature?

Yes

Are all factual statements correct and adequately supported by citations?

Yes

Are the conclusions drawn balanced and justified on the basis of the presented arguments?

Yes

Reviewer Expertise:

Crop plant bioinformatics, variomics, genomics, transcriptomics

I confirm that I have read this submission and believe that I have an appropriate level of expertise to confirm that it is of an acceptable scientific standard, however I have significant reservations, as outlined above.

# Response

**Published:** 2022-05-17

**Authors:** Sebastian Beier

**Affiliations:** Forschungszentrum Jülich GmbH, Germany

Dear Micha M. Bayer,

 thank you for taking the time to review our article and providing valuable feedback on our project. We would like to address your comments below:

- What are the specific differences between plants and other organisms in this context? What requirements does plant data have that other data doesn’t? It would be good to expand on this a little.

There are differences with the precise identification, especially for interoperability and genealogy, as well as the chloroplast genome. We have added some clarification in the introduction part of the manuscript. In contrast to plant species other data domains need a different set of metadata, for example animal sciences in particular need information about the breed and gender of the animal that was genotyped.

- Should a standard such as MIAPPE be established for genotyping experiments/data? This would be a good place to discuss this and perhaps start describing what it could look like.

Such an attempt has indeed been made in the past (Huang et al., 2011, 10.4056/sigs.1994602). However, this was apparently not given much attention by the community, so that no critical mass of users could be generated. MIAPPE itself emphasizes phenotyping and is not currently planning to expand into the genotyping domain. If another attempt is made to introduce a standard for genotyping experiments, MIAPPE can serve as an incubator and provide advice and suggestions, as well as interface with other standards or APIs such as BrAPI.

- The age of pan-genomes is now firmly upon us, and it might be a good idea to add some thoughts on how graph-based genotyping might affect any of the suggestions proposed here - for example, graph-based reference genomes.

Thanks a lot for this question, yes there is indeed a need for a better description about both which reference genome was used for genotyping experiments as well as graph-based genotyping when pan-genomes could be utilized. However, the use of VCF has been discussed in various pan-genome contexts, with the conclusion that VCF is not suitable for structural variants, as it is not appropriate for representing nested or complex variants (Hickey et al., 2020, 10.1186/s13059-020-1941-7). Similar conclusions were reached by Li et al. (2020, 10.1186/s13059-020-02168-z), who found that it is not possible to define coordinates for insertions in VCF, which limits its use for simple variations. In the context of this paper, we believe that this is not the best position to talk about other formats, but to acknowledge the shortcomings of VCF and try to make the format in its current form the most FAIR version it can be. Finally, it should be noted that in future versions of VCF (v4.4 and later) there will be efforts to overcome these shortcomings of the specifications to better represent structural variants in all their complexity (see the following pull requests on github: https://github.com/samtools/hts-specs/pull/465 , https://github.com/samtools/hts-specs/pull/553 ).

- “ *Bioinformaticians who have directly performed the genotyping analyses and thus the creation of the VCF files will consider it a comparatively simple task to enter metadata directly into the file*.” It would be helpful to specify what tools can be used (and how) to add metadata to VCF headers and how this can be done with minimal risk of getting things wrong (e.g. mislabelling samples). Is manual editing of a header and replacing it with bcftools the best we have, or are there tools out there that can achieve this in a more foolproof/refined fashion?

To our knowledge, there is no tool that could do this. One possibility would be that read mapping tools are able to provide metadata on samples and write this data directly into the mapping files.To ensure that the metadata is not lost in the downstream analysis and calculation of all subsequent tools, these metadata fields would need to be known and would not be allowed to be overwritten/edited. In hindsight, this may be too ambitious to implement at this stage and it may make more sense to develop a tool capable of adding this metadata to the final VCF file before submission to public archives. We would like to highlight the role of VCF as a data exchange format in particular, this means that VCFs are in general the result of an analysis pipeline or exported resultset from a database (such as EMBL EVA). Manual maintenance of exchange formats, like JSON or XML-based formats can certainly not be excluded, but should rather be the exception. From our point of view this would mean VCF-generating pipelines should implement necessary components for the user-friendly, correct registration of metadata. We have added a paragraph in the text highlighting the need for appropriate supporting tools or APIs for the validation of VCFs and in particular the VCF metadata.

- It would be beneficial to mention the ##META header lines for defining phenotype metadata that were introduced in the VCF v4.3 revision and to provide some examples of how they could be used. How do they fit into the existing framework of data in EVA and ENSEMBL?

The ##META meta-information of the VCF 4.3 is used to define sample descriptors and not phenotypes in the sense of MIAPPE. They might be used for some of the elements listed in BioSamples checklists, such as the MIAPPE list used by the BioSamples Validator. But our approach was to include only the minimal identification metadata in the VCF (DOIs, ID lists) and rely on BioSamples for a detailed description of the sample. Regarding the ##META field: There is currently a discussion in the VCF community to remove this field completely from the specification (https://github.com/samtools/hts-specs/issues/558#issuecomment-829421610), and basically the trend is more moving towards outsourcing metadata to FAIR archives (like BioSamples). 

- P.7, section “Sample field format”: Please include some detail on what the “registry” entails – is there a standardised way of creating/naming/referring to registries?

Thanks, we clarified this point in the paper.

- P.9, bottom: Please provide a reference and/or URL for ELIXIR in the text here.

We added a recent publication about ELIXIR to the manuscript.

- Figure 2 is an idealised scenario that assumes an institution has both a LIMS and a data steward. Neither is a given – this very much depends on the organisational structure and the levels of funding available to an institution. It would be helpful to have – in addition – an alternative workflow that is more realistic/flexible and describes what can be done when these resources aren’t available.

Genotyping of large panels of genotypes or pan-genome projects depend on a well-structured implementation of the necessary data handling processes according to the research data life cycle to allow quality and efficiency of resources as well as to ensure the long-term assured re-usability of the data. The use of process-oriented database-based solutions starting with material and sample description, the assignment of PUIDs for sequences and material, the machine-readable data processing in sequence laboratories and the ingestion into file storage systems, their registration in in-house databases and their final publication in central repositories, such as EMBL-EBI EVA, is an important task for medium to large institutions. The definition of clear specifications for data exchange formats and APIs provides the necessary framework to allow sufficient freedom for technologies. These can range from open source to commercial solutions that are sufficiently available for the process described. A best practice paper has been referenced accordingly in the manuscript: https://doi.org/10.1093/bib/bbab010 . We see our contribution to this discussion as a standardised workflow that can occur in many different variations. For example, it is indeed a problem that institutions do not have dedicated data stewards or an institution-wide LIMS, so that these sub-aspects either have to be outsourced or interim solutions have been created. In the context of this manuscript, we cannot and do not want to refer to all the possibilities for implementing good scientific practice, but rather provide a stylised best practice guide to be able to map such a workflow to one's own organisation.

- Figure 2: Replace pronouns like “he” with gender-neutral equivalents like “they” or use the passive voice.

We changed this to gender-neutral equivalents within the manuscript.

- Figure 2: The header says “ *…submission of genotypic data to public databases*” but then the legend only mentions the EVA specifically.

We have changed the text in the figure header accordingly to make it clear that this workflow is intended for use with EMBL-EBI repositories.

# Peer review: Reviewer response for version 1

**DOI:** 10.5256/f1000research.120539.r125389

**Published:** 2022-03-02

**Authors:** Boas Pucker, Alenka Hafner

**Affiliations:** 1 Institute of Plant Biology & BRICS, TU Braunschweig, Braunschweig, Germany; 2 Intercollege Graduate Degree Program in Plant Biology, Penn State University, University Park, PA, USA

Beier *et al.* present their recommendations for the inclusion of metadata in VCF files. A defined structure is proposed to make VCF files more suitable for re-use. Suggested fields include file date, bioinformatics source, reference URL, contig, and sample. This opinion paper adds a novel perspective to the area. It is important for advancing FAIR and reproducibility in general if individual formats are analysed in this manner. The suggested metadata standards for VCF seem appropriate for users, with some clarification/additions (see comments). The paper is well and clearly written. The figures help the narrative and provide a quick overview.

**Specific comments:**

1. FAIR principles are not directly discussed in the text despite the abstract/key word emphasis. The term is only mentioned twice in the manuscript. The recommendations presented in this paper would improve many aspects of FAIR in VCF but this is not explicitly discussed. There is mention of individual components but a more direct discussion would be beneficial.
2. Why is gVCF excluded? Are there reasons that prevent the direct transfer of this VCF standard to gVCF?
3. The introduction mentions a few variant calling databases. What about 1001 genomes GMI-MPI vcf ( https://1001genomes.org/data-center.html)? Several variant datasets for *Arabidopsis thaliana* are also hosted on https://jbrowse.arabidopsis.org/. It is only for *Arabidopsis *but considering the importance of this model it should be mentioned that genomic variation data is often hosted on organism-specific databases (which is in itself a problem of format unification). GVM, for example, does not host Arabidopsis VCFs presumably because they are found in their own database.
4. The introduction assumes that the reference genome sequence is supported by Ensembl, but what to do if it is not available?
5. Considering that phenotype standards are the only provided example, it does not seem like the most useful comparison. The data type/collection/prevalence etc. is very different to VCF. More examples would be needed to emphasize shared elements of various standards (for example, SAM/BAM flags).
6. One statement about VCFs should be checked: "one or more data lines". It does not make much sense to share such a file, but the VCF file could be empty i.e. no lines with data.
7. "well-formed" could be explained in more detail.
8. It might be better to use bioinformatics_source to include a complete description of the data processing in the VCF file. Otherwise, there is the risk that data sets cannot be re-used due to broken links. Some data sets might be the result of unpublished protocols hence it will be impossible to link to a publication. For example, the data set and the workflow might be part of the same paper.
9. RefSeq instead of GenBank might be preferentially entered by some groups so this needs to be screened/specified. Again, what happens if the reference genome sequence is not publicly available (yet)? Fig. 2 nicely points out that we must also consider a not-yet-published reference used by a lab to adhere to this standard by registering their reference. It would be good to mention this in the description of the respective field as well.
10. Some assumptions underlying Fig.2 might be too optimistic. The actual sequencing of many projects is conducted by external sequencing providers. Many institutions also lack a LIMS and a "Data Steward". Maybe it would be better to adjust the process in Fig.2 or display an alternative scenario. What would happen if, say, an inexperienced PhD student needs to handle the entire process?
11. "genome" should be replaced by "genome sequence" at several places in manuscript. This is about different assembly version of the same accession so the genome remains the same, but the quality of the representation improves.
12. It would be good to mentioned other examples of VCF containing databases besides EVA and GVM. Even though they are the main ones, one of the biggest issues with metainformation is that you have “straggler” databases in niche fields/organisms that do not adhere to the format.
13. With respect to the tools automatically changing information about variant positions: Can this be monitored in any way? It is not reasonable to expect, e.g. EVA to check this and presumably it is databases that would need to enforce these standards. Would a list of common programs that change this help to avoid issues?
14. Additional meta-information fields:
  a) "findability and interoperability": To reiterate a comment above - reusability (at least, if not also accessibility) would be enhanced by these metadata standards. The impact/implications to VCF FAIR would fit the narrative in here and in the conclusion.
  b) "metainformation lines”: Indeed, and some some suggestions, e.g. from the barley example are needed.
15. "genotypic and other data from plants": What are these other data? Please specify if this would be phenotypic.
16. "Analytical results should be linked out to the respective sample(s) and defined in the context of the study being reported." ... this could be a bit more specific.
17. It does not become clear how the paragraph about BioSample database at EMBL-EBI contributes to the conclusion. Currently, it is only about phenotypic data and a connection to VCF would be helpful.
18. Re-use is a very important aspect of missing/insufficient metadata consequences. It could be emphasised more as a major benefit of adopting this type of VCF standardisation.
19. More specific portals for adoption would be beneficial to mention in the conclusion - should all databases adopt these guidelines, should journals make sure the data referenced in the paper adheres to them, etc.? Enforcement is a major hurdle of adopting metadata (and data) standards that adhere to FAIR (see our paper, Sielemann, Hafner & Pucker, 2020 ^1^).
20. The authors focus on plant VCF and EVA. However, considering these standards are sorely needed in other areas/databases and that VCF formatting should be fairly universal, the authors might want to consider a broader application of these standards. This could be suggested and explored more in the conclusion.
21. It could be helpful to include a sample of the improved VCF as supplementary file.

**Minor comments:**

1. The link to one reference is not correctly formatted: "NCBI Insights, 2017".
2. 'Each contig contains at least the attribute ID, and typically also include length' > "Each contig entry contains at least the attribute ID, and typically also includes length..."

Is the topic of the opinion article discussed accurately in the context of the current literature?

Yes

Are arguments sufficiently supported by evidence from the published literature?

Yes

Are all factual statements correct and adequately supported by citations?

Yes

Are the conclusions drawn balanced and justified on the basis of the presented arguments?

Yes

Reviewer Expertise:

Plant genomics, plant specialized metabolism

We confirm that we have read this submission and believe that we have an appropriate level of expertise to confirm that it is of an acceptable scientific standard, however we have significant reservations, as outlined above.

## References

[1] : The reuse of public datasets in the life sciences: potential risks and rewards. *PeerJ* .2020;8: 10.7717/peerj.9954 e9954 10.7717/peerj.9954 33024631PMC7518187

# Response

**Published:** 2022-05-17

**Authors:** Sebastian Beier

**Affiliations:** Forschungszentrum Jülich GmbH, Germany

Dear Boas Pucker and Alenka Hafner,

 thank you for taking the time to review our article and providing valuable feedback on our project. We would like to address your comments below:

 1. FAIR principles are not directly discussed in the text despite the abstract/key word emphasis. The term is only mentioned twice in the manuscript. The recommendations presented in this paper would improve many aspects of FAIR in VCF but this is not explicitly discussed. There is mention of individual components but a more direct discussion would be beneficial.

 We added explicit FAIR principles discussion in the discussion to clearly state the improved Findability, Reusability and Interoperability.

 2. Why is gVCF excluded? Are there reasons that prevent the direct transfer of this VCF standard to gVCF?

 We added some remarks, showing these recommendations can be directly transferred to gVCF. There is nothing that would exclude the use of our recommendations with gVCF.

 3. The introduction mentions a few variant calling databases. What about 1001 genomes GMI-MPI vcf ( https://1001genomes.org/data-center.html )? Several variant datasets for *Arabidopsis thaliana* are also hosted on https://jbrowse.arabidopsis.org/ . It is only for *Arabidopsis *but considering the importance of this model it should be mentioned that genomic variation data is often hosted on organism-specific databases (which is in itself a problem of format unification). GVM, for example, does not host Arabidopsis VCFs presumably because they are found in their own database.

 We added some remarks regarding this in the manuscript.

 4. The introduction assumes that the reference genome sequence is supported by Ensembl, but what to do if it is not available?

 In the case where the reference genome sequence is not yet in Ensembl the VCF cannot be displayed (within Ensembl). However in such cases Ensembl would try to prioritize adding the missing genome if it is in the public archives. This should not be confused with EVAs requirement of needing a genome assembly supported by INSDC.

 5. Considering that phenotype standards are the only provided example, it does not seem like the most useful comparison. The data type/collection/prevalence etc. is very different to VCF. More examples would be needed to emphasize shared elements of various standards (for example, SAM/BAM flags).

 It is true that MIAPPE, and thus the phenotyping standard, was not intended to characterize genotyping analyses or VCF files. It has been used to enable interoperability with genotyping dataset through shared objects and IDs. It is also true that in order to create a VCF file, there must be one or more mapping files (SAM/BAM) that record the differences and similarities between the genotypes under study and the reference genome assembly. Since there are a large number of different workflows to create a VCF file, our intention was to make the final result FAIR and not necessarily cover all intermediate steps with our suggestions. What all genotyping experiments have in common is that samples are obtained from physical material that is currently poorly described. It is precisely this point that is better addressed and makes it possible to link different experiments and analyses based on the material used with the recommendations presented here. MIAPPE offers a well-designed framework for this and is also ideally suited for the plant domain.

 6. One statement about VCFs should be checked: "one or more data lines". It does not make much sense to share such a file, but the VCF file could be empty i.e. no lines with data.

 We agree that sharing such a file would not make much sense, still it would be a perfectly valid VCF file according to the specifications. This wording is taken directly from the specifications.

 7. "well-formed" could be explained in more detail.

 We have adjusted the text to make this clearer.

 8. It might be better to use bioinformatics_source to include a complete description of the data processing in the VCF file. Otherwise, there is the risk that data sets cannot be re-used due to broken links. Some data sets might be the result of unpublished protocols hence it will be impossible to link to a publication. For example, the data set and the workflow might be part of the same paper.

 This is a valid concern, but we cannot see that embedded documentation will ensure a sustainable, comprehensive and FAIR documentation of a workflow that enables a re-processing in 10 years. For example, used software needs to be referred to using PUIDs or links. Here, we could also face broken links. If mentioned software or scripts cannot be resolved anymore, VCF embedded documentation could become ambiguous too. To get around this, a container (RO-Crate, Docker, Singularity, etc.) is one of the only solutions that can remedy this and make both the workflow and the data accessible to users. It should be noted here, however, that there are some scenarios where the disclosure of all programmes, scripts or data is not wanted or legally possible. However, until containers are common practice, explaining the workflow in as much detail as possible is a good start. There are some approaches that map this in a more structured way, such as Common Workflow Language (CWL, https://doi.org/10.1038/sdata.2018.118 ). In general, however, it should be said that here the workflows can also be referenced as DOI, which is fully compliant with our recommendations.

 9. RefSeq instead of GenBank might be preferentially entered by some groups so this needs to be screened/specified. Again, what happens if the reference genome sequence is not publicly available (yet)? Fig. 2 nicely points out that we must also consider a not-yet-published reference used by a lab to adhere to this standard by registering their reference. It would be good to mention this in the description of the respective field as well.

 The complete described pipeline builds upon deposition of genotyping information at EVA, which needs a published genome assembly accession number. That is one of the reasons Fig. 2 also tries to highlight that the genome assembly needs to be deposited and an accession number received before being able to proceed to the next step. We updated the text to make this more clear. In addition, the trend in genome sequencing and assembly is for both the read data and the assembly sequence to be published early, making it less likely in the future that the genome sequence will not be publicly available.

 10. Some assumptions underlying Fig.2 might be too optimistic. The actual sequencing of many projects is conducted by external sequencing providers. Many institutions also lack a LIMS and a "Data Steward". Maybe it would be better to adjust the process in Fig.2 or display an alternative scenario. What would happen if, say, an inexperienced PhD student needs to handle the entire process?

 Please have a look at our response to Micha Bayer’s comment referring to Figure 2. As additional clarification and help for more inexperienced scientists we have published a stepwise guide on how to submit data using the recommendations in this manuscript in the FAIR Cookbook under recipe https://w3id.org/faircookbook/FCB061.

 11. "genome" should be replaced by "genome sequence" at several places in manuscript. This is about different assembly version of the same accession so the genome remains the same, but the quality of the representation improves.

 Indeed, a good catch. We updated the manuscript to be more precise.

 12. It would be good to mentioned other examples of VCF containing databases besides EVA and GVM. Even though they are the main ones, one of the biggest issues with metainformation is that you have “straggler” databases in niche fields/organisms that do not adhere to the format.

 In this paper, we focused on the use case of EMBL-EBI submissions and their use of VCF in collaboration with BioSamples and EVA. We are not aiming at an extensive review of all possible VCF submissions and publishers. We added some remarks in the discussion regarding adoption of the metadata recommendations in the broader community.

 13. With respect to the tools automatically changing information about variant positions: Can this be monitored in any way? It is not reasonable to expect, e.g. EVA to check this and presumably it is databases that would need to enforce these standards. Would a list of common programs that change this help to avoid issues?

 We are not aware of any programs that change the position or other characteristics of variants, but programs such as PLINK are known to define the variant within a genotyping study with the major allele as the reference and the minor allele as the alternative, regardless of the base present in the reference genome assembly. However, PLINK has an option to use the reference allele, and EVA often asks to correct this at the time of submission. EVA also consistently validates each VCF file that is submitted to the archive. Validation compares the reference allele to the reference genome sequence, most likely detecting misplaced variants. It is technically possible for misplaced variants to still match the reference, but in practice this method will detect most of these errors. A list of common programs that work in a similar way to PLINK would be very welcome. However, we do not believe that this would completely avoid this problem, since such a list is not easy to maintain (based on new versions, programs, workflows) and could lull the user into a false sense of security if other programs were used. We would therefore rather urge caution and diligence when using programs that interact with VCF files.

 14. Additional meta-information fields:

 a) "findability and interoperability": To reiterate a comment above - reusability (at least, if not also accessibility) would be enhanced by these metadata standards. The impact/implications to VCF FAIR would fit the narrative in here and in the conclusion.

 b) "metainformation lines”: Indeed, and some some suggestions, e.g. from the barley example are needed.

 a) We added reusability in the listing and expanded on the FAIRness of data in the discussion part. b) Information, such as further metadata about the person who analysed the data or collected the plant material and the prevailing environmental conditions (e.g. contact information, tools used, temperature or humidity), would be examples of additional metainformation fields that were not explicitly recommended by us, but in some scenarios make sense to include additionally.

 15. "genotypic and other data from plants": What are these other data? Please specify if this would be phenotypic.

 Since the sample description would be uniform, this could be applied to all kinds of different data. This includes phenotypic data but also other -omics layers, like transcriptomic data, metabolomic data and so forth.

 16. "Analytical results should be linked out to the respective sample(s) and defined in the context of the study being reported." ... this could be a bit more specific.

 We have updated the text to be more precise.

 17. It does not become clear how the paragraph about BioSample database at EMBL-EBI contributes to the conclusion. Currently, it is only about phenotypic data and a connection to VCF would be helpful.

 We have updated the wording to clarify that the plant metadata stored at BioSamples does not necessarily have to be of phenotypic origin.

 18. Re-use is a very important aspect of missing/insufficient metadata consequences. It could be emphasised more as a major benefit of adopting this type of VCF standardisation.

 We agree with this statement and have added this throughout the manuscript.

 19. More specific portals for adoption would be beneficial to mention in the conclusion - should all databases adopt these guidelines, should journals make sure the data referenced in the paper adheres to them, etc.? Enforcement is a major hurdle of adopting metadata (and data) standards that adhere to FAIR (see our paper, Sielemann, Hafner & Pucker, 2020).

 We agree with the concerns raised in Sielemann, Hafner & Pucker, 2020, and one of the solutions they offer is improvement of metadata standard which is what this paper tries to accomplish. 

 Enforcing these new metadata standards on many underfunded databases is unlikely. Adoption of new standards is always tricky and takes time but we believe the best way for that is to enforce it in a central point like upon submission to the archives. 

 Alternatively, journals could be approached to enforce compliance with the recommendations. However, one obstacle would be convincing not just one institution but many different publishers to do so, which is very time-consuming. In our view, critical mass is more likely to be achieved through community engagement via larger organisations (such as DivSeek, AgBioData, Australian BioCommons, etc.). We have started to do this and there is overall positive feedback and early signs of acceptance within these organisations.

 20. The authors focus on plant VCF and EVA. However, considering these standards are sorely needed in other areas/databases and that VCF formatting should be fairly universal, the authors might want to consider a broader application of these standards. This could be suggested and explored more in the conclusion.

 Indeed, we are very interested in bringing these ideas and recommendations to other groups. In particular we have been in discussions with scientists in the animal field to work on an adaptation to their field. 

 21. It could be helpful to include a sample of the improved VCF as supplementary file.

 We have uploaded an example of this workflow to EVA and highlighted this in the manuscript. The VCF has been validated and accessioned successfully by EVA. The Study is now available on the EVA website at https://www.ebi.ac.uk/eva/?eva-study=PRJEB51851 .

**Minor comments:**

 1. The link to one reference is not correctly formatted: "NCBI Insights, 2017".

 We have changed the reference to the EVA paper published last year where this was stated. 

 2. 'Each contig contains at least the attribute ID, and typically also include length' > "Each contig entry contains at least the attribute ID, and typically also includes length..."

 Changed this accordingly in the manuscript.
